# Compressed sensing 3D T2WI radiomics model: improving diagnostic performance in muscle invasion of bladder cancer

**DOI:** 10.1186/s12880-024-01318-0

**Published:** 2024-06-17

**Authors:** Shuo Li, Zhichang Fan, Junting Guo, Ding Li, Zeke Chen, Xiaoyue Zhang, Yongfang Wang, Yan Li, Guoqiang Yang, Xiaochun Wang

**Affiliations:** 1 Department of Medical Imaging, Shanxi Medical University, Taiyuan, 030001 Shanxi P.R. China; 2 Department of Radiology, The First Hospital of Shanxi Medical University, No.85 Jiefang South Road, Taiyuan, 030001 Shanxi Province P.R. China

**Keywords:** Magnetic resonance imaging, Compressed sensing, Bladder cancer, Muscle invasion, Radiomics

## Abstract

**Background:**

Preoperative discrimination between non-muscle-invasive bladder cancer (NMIBC) and the muscle invasive bladder cancer (MIBC) is a determinant of management. The purpose of this research is to employ radiomics to evaluate the diagnostic value in determining muscle invasiveness of compressed sensing (CS) accelerated 3D T2-weighted-SPACE sequence with high resolution and short acquisition time.

**Methods:**

This prospective study involved 108 participants who underwent preoperative 3D-CS-T2-weighted-SPACE, 3D-T2-weighted-SPACE and T2-weighted sequences. The cohort was divided into training and validation cohorts in a 7:3 ratio. In the training cohort, a Rad-score was constructed based on radiomic features selected by intraclass correlation coefficients, pearson correlation coefficient and least absolute shrinkage and selection operator . Multivariate logistic regression was used to develop a nomogram combined radiomics and clinical indices. In the validation cohort, the performances of the models were evaluated by ROC, calibration, and decision curves.

**Results:**

In the validation cohort, the area under ROC curve of 3D-CS-T2-weighted-SPACE, 3D-T2-weighted-SPACE and T2-weighted models were 0.87(95% confidence interval (CI):0.73-1.00), 0.79(95%CI:0.63–0.96) and 0.77(95%CI:0.60–0.93), respectively. The differences in signal-to-noise ratio and contrast-to-noise ratio between 3D-CS-T2-weighted-SPACE and 3D-T2-weighted-SPACE sequences were not statistically significant(*p* > 0.05). While the clinical model composed of three clinical indices was 0.74(95%CI:0.55–0.94) and the radiomics-clinical nomogram model was 0.88(95%CI:0.75-1.00). The calibration curves confirmed high goodness of fit, and the decision curve also showed that the radiomics model and combined nomogram model yielded higher net benefits than the clinical model.

**Conclusion:**

The radiomics model based on compressed sensing 3D T2WI sequence, which was acquired within a shorter acquisition time, showed superior diagnostic efficacy in muscle invasion of bladder cancer. Additionally, the nomogram model could enhance the diagnostic performance.

**Supplementary Information:**

The online version contains supplementary material available at 10.1186/s12880-024-01318-0.

## Introduction

Bladder cancer (BCa) is a disease with a high rate of incidence, recurrence and mortality, and one of the most common malignant tumors of the urinary system in elderly men [1–3]. The majority of tumors are urothelial cell carcinomas, which can be categorized based on muscle invasiveness into non-muscle invasive bladder cancer (NMIBC) and muscle invasive bladder cancer (MIBC). Additionally, they can be classified into low- and high-grade lesions based on histology [4, 5]. The muscle invasiveness is the major consideration for treatment decision of BCa. NMIBC generally is treated with transurethral resection (TURBT), and has a supplement of chemotherapy. While MIBC need intensive treatment like the radical cystectomy (RC) due to the aggressive tumor [6–9]. Recent studied have proved that adjuvant chemotherapy had great performance in increasing overall survival of MIBC [10, 11].

MRI plays an important role in the preoperative diagnosis of BCa. The T2-weighted sequence is an essential component of multiparametric MRI protocols for evaluation muscle invasiveness in BCa [12]. Compared to the conventional T2-weighted sequence, the 3D-T2-weighted-SPACE sequence has higher resolution for imaging anatomical structure of female pelvis, so that it can reflect muscle invasiveness of tumor better [13, 14]. And what’s more, the high-resolution image containing tiny voxels and elaborate grayscales could provide more comprehensive information for quantitative analysis of radiomics [15]. But the scanning time of 3D-T2-weighted-SPACE is still too long for clinical practice. Adequate bladder distension is necessary for clearly imaging the bladder wall but also causes discomfort to patients. However, due to bowel peristalsis, motion and susceptibility artifacts are often observed in bladder MR images. Therefore, shortening acquisition times had benefits in decreasing patients’ discomfort and improving image quality [16]. Through k-space undersampling, compressed sensing (CS) technology reduces redundant scanning data and accelerate acquisition [17]. Thus, 3D-CS-T2-weighted-SPACE sequence could maintain the image quality under an acceptable acquisition time.

At present, the gold standard for clinical determination of muscular invasiveness of BCa is cystoscopy biopsy, but due to the spatiotemporal heterogeneity of tumors, differences in transurethral biopsy techniques may lead to misdiagnosis [10, 18]. It was reported that 20–80% of lesions were incorrectly staged compared with postoperative pathological staging because of variations in performing cystoscopy biopsy [19–21]. Therefore, there is quite necessary to find a noninvasive diagnostic tool to achieve accurate discrimination between NMIBC and MIBC in clinical practice. Radiomics, a method involving the extraction of extensive quantitative image features from medical imaging, has become increasingly prominent in cancer research in recent years [22–24]. Unlike traditional subjective evaluations of imaging characteristics, radiomics offers an objective approach and can capture high-dimensional imaging features that may correlate with intratumor heterogeneity [25, 26]. Previous studies have utilized CT/MRI-based radiomics signatures to predict various biological behaviors in BCa, such as muscle-invasive status, lymph node metastasis, tumor stage, prognosis, and treatment response [26–29]. The high-resolution thin-section 3D-SPACE sequence possesses the capability to capture a wealth of tumor information, facilitating a comprehensive and objective portrayal of tumor heterogeneity.

Therefore, the purpose of our study is to explore whether the model extracted from 3D-CS-T2-weighted-SPACE sequence still has good diagnostic value for detecting muscle invasion of bladder cancer while reducing time.

## Methods

This prospective research was approved by our institutional review board, and informed consent was obtained from all subjects.

### Patients

A total of 108 patients (including 89 males and 19 females, aged 20–86 years [65 ± 11]) with pathological confirmed BCa were prospectively analyzed between June 10, 2022, and March 20, 2023, at the First Hospital of Shanxi Medical University. Their clinical and imaging data were collected.

The inclusion criteria included the following: (1) Patients with suspicious bladder lesions were identified using multimodal magnetic resonance imaging before surgery; (2) Transurethral resection or radical resection of bladder cancer was performed within 1 month, confirming the diagnosis of bladder cancer.

**Fig. 1:**
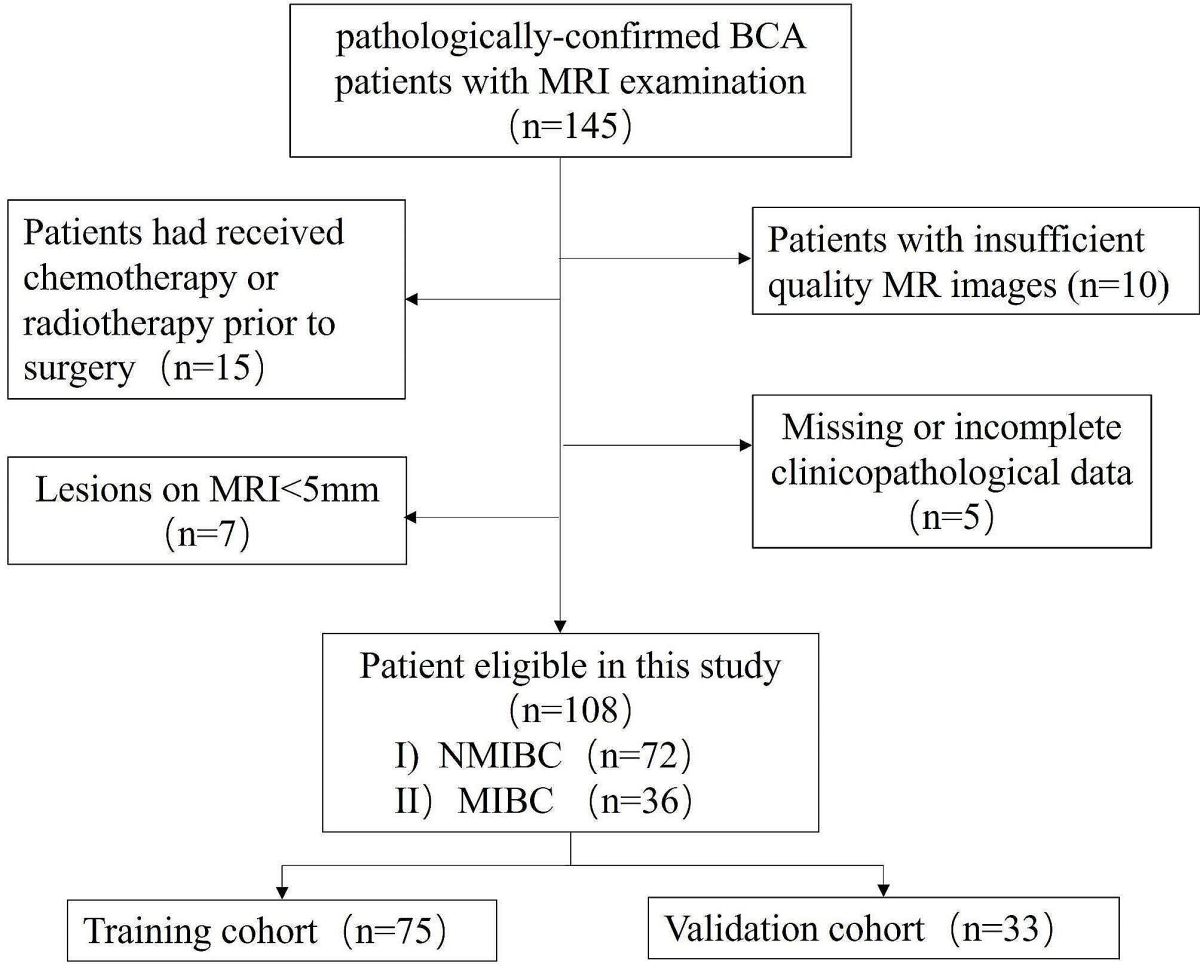
The patient deletion flow chart

The exclusion criteria included the following: (1) Patients had received chemotherapy or radiotherapy prior to surgery; (2) Lesions with poor image quality and serious motion artifacts; (3) Missing or incomplete clinicopathological data. (4) Lesions on MRI < 5 mm **(**Fig. 1**)**.

### MR Image acquisition

**Fig. 2:**
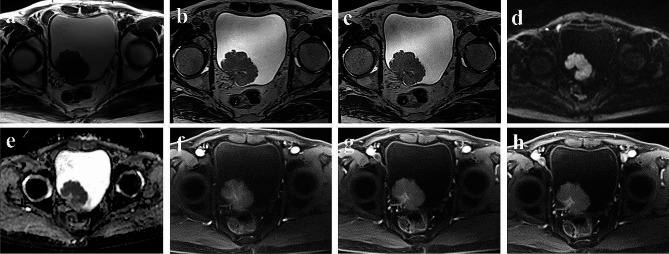
**(a-h)** display a bladder tumor located at the right ureteral orifice, pathologically diagnosed as non-muscle invasion. Axial T2WI **(a)**, T2-weighted-SPACE **(b)**, CS-T2-weighted-SPACE **(c)**, DWI **(d)**, ADC **(e)**, arterial phase **(f)**, intravenous phase (g), and delayed phase **(h)**

**Fig. 3:**
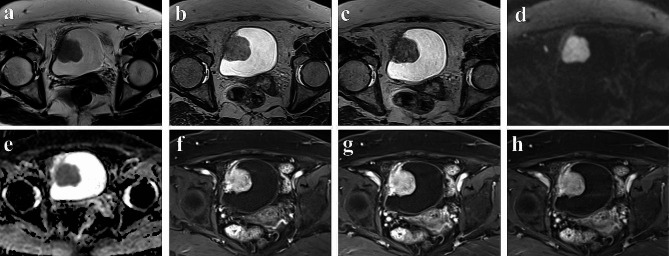
**(a-h)** display a bladder tumor located in the right wall of the bladder, pathologically diagnosed as muscle invasion. Axial T2WI **(a)**, T2-weighted-SPACE **(b)**, CS-T2-weighted-SPACE **(c)**, DWI **(d)**, ADC **(e)**, arterial phase **(f)**, intravenous phase **(g)** and delayed phase **(h)**

All patients were scanned by a 3.0T MR scanner (MAGNETOM Vida: Siemens, Erlangen, Germany). The scanning range covered the pelvis (bladder), and the scanning sequence included conventional axial T2-weighted FSE sequences, 3D-T2-weighted-SPACE and 3D-CS-T2-weighted-SPACE (with acceleration factor 6.0). T2-weighted image scanning parameters were: repetition time (TR) = 4170 msec; echo time (TE) = 106 msec; slice thickness = 6 mm; matrix 384 × 384, FOV = 370 × 370 mm, Conventional axial, sagittal and coronal T2 TSE [6-mm section thickness] required acquisition times of 1 min 53 s, 1 min 42 s and 1 min 48 s, respectively (total 5 min 23 s). 3D-T2-weighted-SPACE image scanning parameters were: TR = 1700 msec; TE = 100 msec, slice thickness = 1 mm; matrix was 448 × 448, FOV = 224 × 224 mm, Acquisition time 4 min 08 s. 3D-CS-T2-weighted-SPACE scanning parameters were: TR = 1600 msec; TE = 95 msec; slice thickness = 0.9 mm; matrix 512 × 512, FOV = 230 × 230 mm, Acquisition time 3 min 58 s. Figures 2 and 3 shows the imaging performance of two patients with bladder cancer on MRI.

### Region of interest (ROI) delineation

**Fig. 4:**
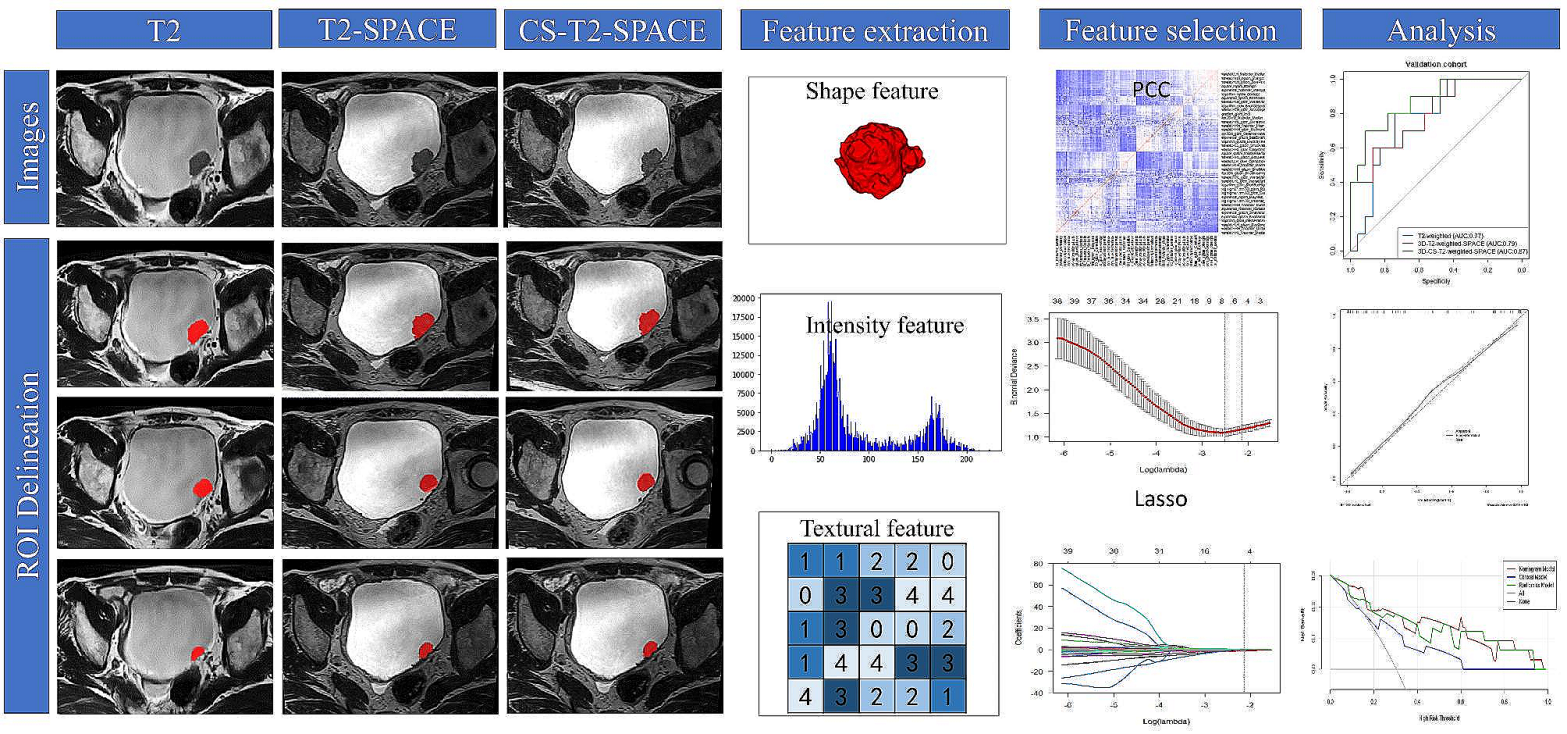
Radiomics workflow

Figure 4 shows the radiomics workflow. One radiologist (Y.L. with over 7 years of experience in bladder MRI reading), who was blinded to the pathological results, segmented the region of interest (ROI). For each BCa patient, the boundaries of the tumor were drawn on T2-weighted axial image slices using ITK-SNAP software (version 3.8.0; http://itk-snap.org) to obtain the volume of interest (VOI) of the bladder tumor. The areas of tumor stalk, blood vessels, and necrosis were excluded. Multiple lesions, based on VI-RADS, the highest scoring lesion was selected as the index-lesion. After 30 days, the VOIs of bladder tumors from 30 randomly selected patients were repeatedly drawn by the same radiologist and another radiologist (Y.F.W with 6 years of experience in body MR) to calculate the intraclass correlation coefficients (ICC). For feature extraction, ICC values greater than 0.75 were considered to indicate good consistency.

### Radiomics feature extraction

We categorized the imaging features into four classes, namely shape and size-based features, image intensity features (first-order features), textural features, and advanced features. A total of 5343 radiomics features were extracted from axial T2-weighted, 3D-T2-weighted-SPACE, and 3D-CS-T2-weighted-SPACE sequences, with 1781 features per sequence. Before feature screening, each feature was normalized using a Z-score. The open-source software FAE (http://github.com/salan668/FAE, accessed on 20 September 2020), based on the Pyradiomics package (https://github.com/Radiomics/Pyradiomics, accessed on 20 September 2020), was used for extracting radiomic features.

### Radiomics feature selection and radiomics model construction

First, features with ICC ≥ 0.75 were utilized for subsequent feature selection. Pearson correlation coefficients (PCC) were then employed for dimensionality reduction, with a threshold set at 0.90. If the coefficient exceeded 0.90, one of the features was randomly eliminated. The synthetic minority oversampling technique (SMOTE) method addressed the imbalance between positive and negative samples. Subsequently, the least absolute shrinkage and selection operator (LASSO) algorithm was employed for feature selection using 5-fold cross-validation. The process is depicted in Supporting Information Fig. S1. Using the same method, optimal radiomics features were identified from two other sequences. We built the radiomics model using logistic regression, wherein the radiomics score (Rad-score) was obtained by summing the selected features weighted by their coefficients in the best model. All differentiation classifiers were developed on the training cohort and validated on the validation cohort.

### Clinical model and Nomogram Development

In the training cohort, clinical and imaging characteristics were included in the univariate analysis and multivariate logistic regression analysis to identify major clinical risk factors predictive of MIBC and to construct a clinical model (Table 1). Multivariate Logistic regression was performed on the independent clinical risk indices and Rad-score to construct a radiomic-clinical nomogram [9].

**Table 1:** Results of Univariable and Multivariable Analysis of Clinic-Radiological Characteristics

Clinical and Radiological Characteristics		Univariable Analysis			Multivariable Analysis	
	OR	95%CI	*P*	OR	95%CI	*P*
Age	1	0.96–1.04	0.97	-	-	-
BMI	1	0.89–1.13	0.944	-	-	-
Gender	0.86	0.29–2.56	0.78	-	-	-
Smoke	0.92	0.43–2.01	0.843	-	-	-
Tumor size	0.17	0.07–0.41	< 0.001	0.22	0.08–0.56	0.002
Multiple lesions	0.28	0.12–0.65	0.003	0.35	0.14–0.89	0.028
Total cholesterol	1.12	0.72–1.73	0.627	-	-	-
Triglyceride	1.05	0.62–1.8	0.844	-	-	-
High-density lipoprotein	2.65	0.58–12.21	0.21	-	-	-
Low-density lipoprotein	0.87	0.5–1.53	0.628	-	-	-
Frequent urination	1.31	0.47–3.65	0.604	-	-	-
Urinary urgency	1.11	0.46–2.67	0.822	-	-	-
Odynuria	0.79	0.31–2.04	0.631	-	-	-
Urinary incontinence	1	0.27–3.69	1	-	-	-
Hematuria	0.06	0.01–0.47	0.007	0.08	0.01–0.68	0.021

NOTE: BMI, Body Mass Index; CI, confidence interval

### Model validation and evaluation

The model’s performance was evaluated using the area under the receiver operating characteristic (ROC) curve (AUC), which served as the primary performance metric for predicting MIBC. Accuracy, sensitivity, specificity, positive predictive value (PPV), and negative predictive value (NPV) were calculated based on the maximum Youden index threshold. Decision curve analysis (DCA) and calibration curves were employed to evaluate the performance of the nomogram.

### Statistical analysis

Statistical analysis was performed using R version 4.1.3 (https://www.r-project.org/) or SPSS 26.0. The measurement data were tested for normality, and those that conformed to a normal distribution were expressed as (mean ± standard deviation), while those not conformed to a normal distribution were expressed as the median (upper and lower quartiles). Comparisons of measurement data were performed using the independent samples t-test (for normally distributed and chi-squared) or the Mann-Whitney U test (for skewed distribution or chi-squared). The count data were expressed as examples, and the χ2 test was used for group comparisons. *P* values < 0.05 were regarded as statistically significant. The DeLong test was used to compare the AUC of different models.

### Quantitative analysis of the images

The ROI location was selected on the tumor and fat of the 3D-T2-weighted-SPACE sequence and then reproduced on the 3D-CS-T2-weighted-SPACE sequence. The formulas for calculating the signal-to-noise ratio (SNR) and contrast-to-noise ratio (CNR) are as follows:

$$\begin{gathered}SNR = SItissue/SDtissue,{\text{ }}CNR \hfill \\\,\,\,\,\,\,\,\,\,\,\, = |\left( {SItissu1 - SItissue2} \right)/\surd (SDtissue{1^2} + SDtissue{2^2})| \hfill \\ \end{gathered}$$

Here, SI and SD represent the average signal intensity and signal standard deviation of the region of interest (ROI) in the tumor and fat region. In our calculations, we utilized the tissue standard deviation instead of background noise as the background standard deviation, aiming to avoid inconsistencies in accelerated sparse regions. SNR and CNR for images were compared using the Wilcoxon signed-rank sum test.

## Results

### Patient population

In this study, 108 patients with BCa were randomly divided into two groups: the training cohort (75 cases) and the validation cohort (33 cases). The clinical characteristics of the training and validation cohorts, including age, sex, smoking status, tumor size, number of tumors, BMI value, total cholesterol levels, triglyceride levels, high-density lipoprotein levels, low-density lipoprotein levels, T staging based on MRI images, etc., were listed in Table 2. There were no significant differences in clinical characteristics between the two cohorts, indicating that the allocation of patients were reasonable and had no significant effect on the subsequent radiomics analysis.

**Table 2:** Baseline Demographics of the BCa Patients

Clinical and Radiological Characteristics	Training cohort (*n* = 75)	Validation cohort (*n* = 33)	*P* value
Age (years)	65士11	67士11	0.335
Gender (No (%))			0.229
Male	64 (85.3)	25 (75.8)	
Female	11 (14.7)	8 (24.2)	
Smoke (No (%))			0.05
Yes	38 (50.7)	10 (30.3)	
No	37 (49.3)	23 (69.7)	
Size (No (%))			0.919
<3 cm	53 (70.7)	23 (69.7)	
≥3 cm	22 (29.3)	10 (30.3)	
Multiple (No (%))			0.892
Yes	24(32)	11(33.3)	
No	51(68)	22(66.7)	
BMI value (kg/m2)	23.80士3.16	23.72士3.46	0.908
Total cholesterol (mmol/L)	4.32土0.94	4.24士0.86	0.673
Triglyceride (mmol/L)	1.23 (0.88–1.67)	1.24 (0.91–1.68)	0.952
High-density lipoprotein (mmol/L)	1.11 (0.92–1.28)	1.11 (0.97–1.21)	0.971
Low-density lipoprotein (mmol/L)	2.76 (2 16 − 3 32)	2.83 (2.12–3.25)	0.603
Frequent urination (No (%))			0.314
Yes	14 (18.7)	9 (27.3)	
No	61 (81.3)	24 (72.7)	
Urinary urgency (No (%))			0.938
Yes	21 (28)	9 (27.3)	
No	54 (72)	24 (72.7)	
Odynuria (No (%))			0.455
Yes	16 (21.3)	5 (15.2)	
No	59 (78.7)	28 (84.8)	
Urinary incontinence (No (%))			0.719
Yes	7 (9.3)	2 (6.1)	
No	68 (90.7)	31 (93.9)	
Hematuria (No (%))			0.135
Yes	60 (80)	22 (66.7)	
No	15 (20)	11 (33.3)	
Pathologic stage (No (%))			0.658
T < 2	51 (68)	21 (63.6)	
T ≥ 2	24 (32)	12 (36.4)	

### Construction and evaluation of Radiomics Model

Firstly, we extracted 1781 features from each sequence. After ICC consistency analysis, we selected features with ICC ≥ 0.75 for the three sequences, namely T2-weighted 1495, 3D-T2-weighted-SPACE 1571, and 3D-CS-T2-weighted-SPACE 1608. Then, to remove redundant features, after PCC analysis, we left 320, 252 and 247 features. Finally, the T2-weighted, 3D-T2-weighted-SPACE and 3D-CS-T2-weighted-SPACE models were constructed using the LASSO logistic regression algorithm, after feature screening, we retained 3, 3 and 5 features, respectively. The specific characteristics, corresponding coefficients, and intercept are reported in Supporting Information Table S1.

**Fig. 5:**
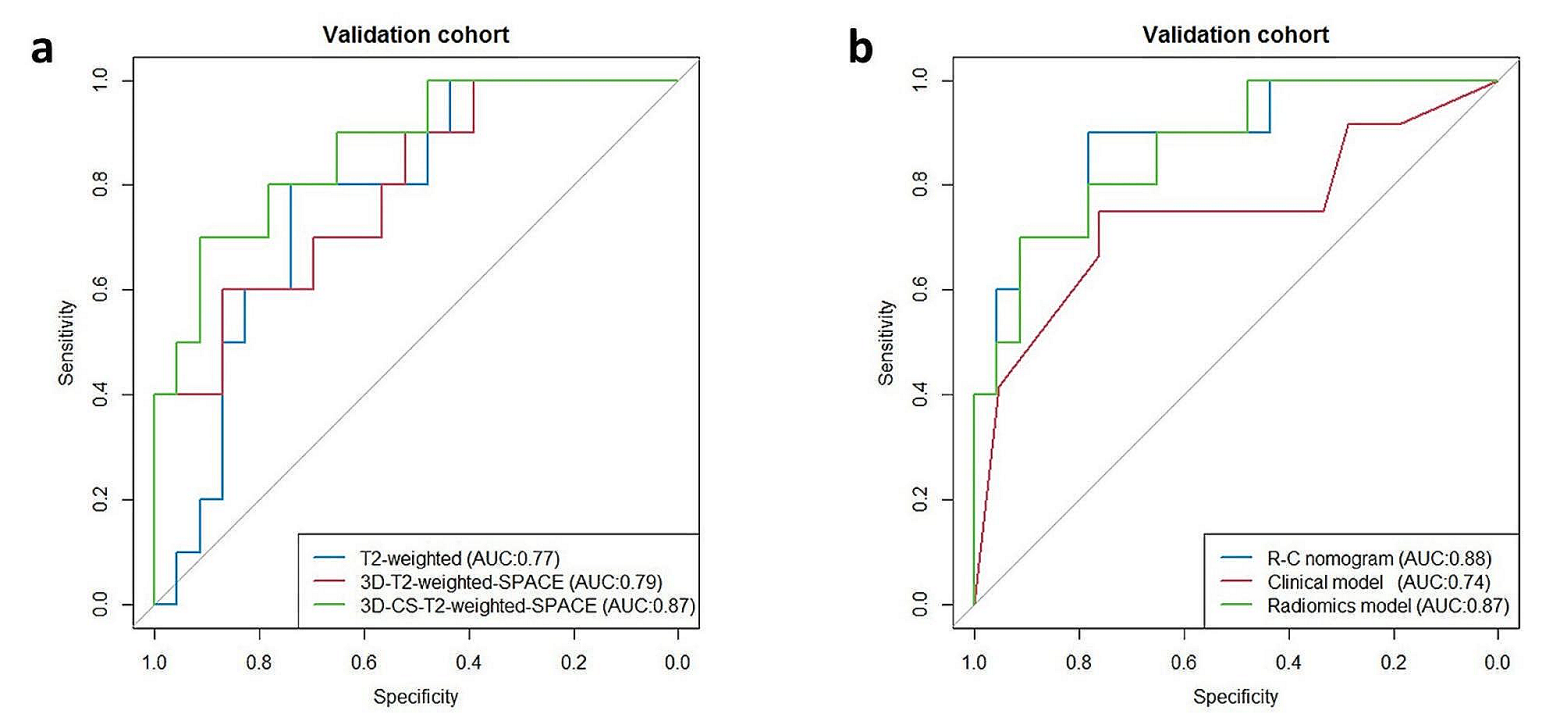
ROC curves of 3 radiomics models **(a)** and using Clinical model, Radiomics model and Radiomics-Clinical combined model **(b)** to predicting the MIBC in the validation cohorts

The accuracy, sensitivity, specificity, and AUC analysis results from the three sequences are shown and compared in Table 3. The model based on the 3D-CS-T2-weighted-SPACE obtained the highest AUC (0.87, 95%CI: 0.73-1.00) among the three different sequence models. The AUC of the model based on 3D-T2-weighted-SPACE was similar to the model based on conventional T2-weighted and slightly better than the T2-weighted model (AUC = 0.79, AUC = 0.77). The ROC curves are shown in Fig. 5.

**Table 3:** Performance of clinical and radiomics models

	AUC (95% CI)	accuracy (95% CI)	specificity (95% CI)	sensitivity (95% CI)	npv (95% CI)	ppv (95% CI)
Training cohort						
T2-weighted	0.87 (0.77–0.95)	0.81 (0.72–0.89)	0.76 (0.73–0.86)	0.92 (0.79–0.98)	0.95 (0.90–0.99)	0.65 (0.51–0.75)
3D-T2-weighted-SPACE	0.87 (0.78–0.95)	0.80 (0.71–0.87)	0.78 (0.65–0.87)	0.83 (0.70–0.93)	0.91 (0.79–0.97)	0.65 (0.51–0.76)
3D-CS-T2-weighted-SPACE	0.89 (0.79–0.96)	0.84 (0.75–0.92)	0.90 (0.76–0.97)	0.71 (0.57–0.82)	0.87 (0.73–0.95)	0.77 (0.67–0.90)
Clinical model	0.80 (0.72–0.89)	0.77 (0.58–0.85)	0.73 (0.58–0.84)	0.82 (0.69–0.91)	0.80 (0.66–0.91)	0.75 (0.62–0.86)
R-C nomogram model	0.95 (0.91–0.99)	0.90 (0.83–0.95)	0.92 (0.81–0.98)	0.88 (0.77–0.96)	0.89 (0.77–0.96)	0.92 (0.80–0.98)
Validation cohort						
T2-weighted	0.77 (0.60–0.93)	0.76 (0.58–0.89)	0.74 (0.52–0.90)	0.80 (0.64–0.90)	0.89 (0.64–0.97)	0.57 (0.31–0.83)
3D-T2-weighted-SPACE	0.79 (0.63–0.96)	0.79 (0.63–0.92)	0.87 (0.76–0.97)	0.60 (0.48–0.83)	0.83 (0.56–0.95)	0.67 (0.37–0.91)
3D-CS-T2-weighted-SPACE	0.87 (0.73-1.00)	0.82 (0.74–0.90)	0.83 (0.74–0.91)	0.80 (0.58–0.90)	0.90 (0.65–0.96)	0.67 (0.37–0.90)
Clinical model	0.74 (0.55–0.94)	0.76 (0.58–0.90)	0.76 (0.52–0.92)	0.75 (0.45–0.95)	0.84 (0.56–0.94)	0.64 (0.35–0.86)
R-C nomogram model	0.88 (0.75-1.00)	0.82 (0.65–0.93)	0.78 (0.56–0.93)	0.90 (0.55-1.00)	0.95 (0.74-1.00)	0.64 (0.35–0.87)

NOTE: npv, negative predictive value; ppv, positive predictive value

### Clinical nomogram with radiomics model and clinical Model

**Fig. 6:**
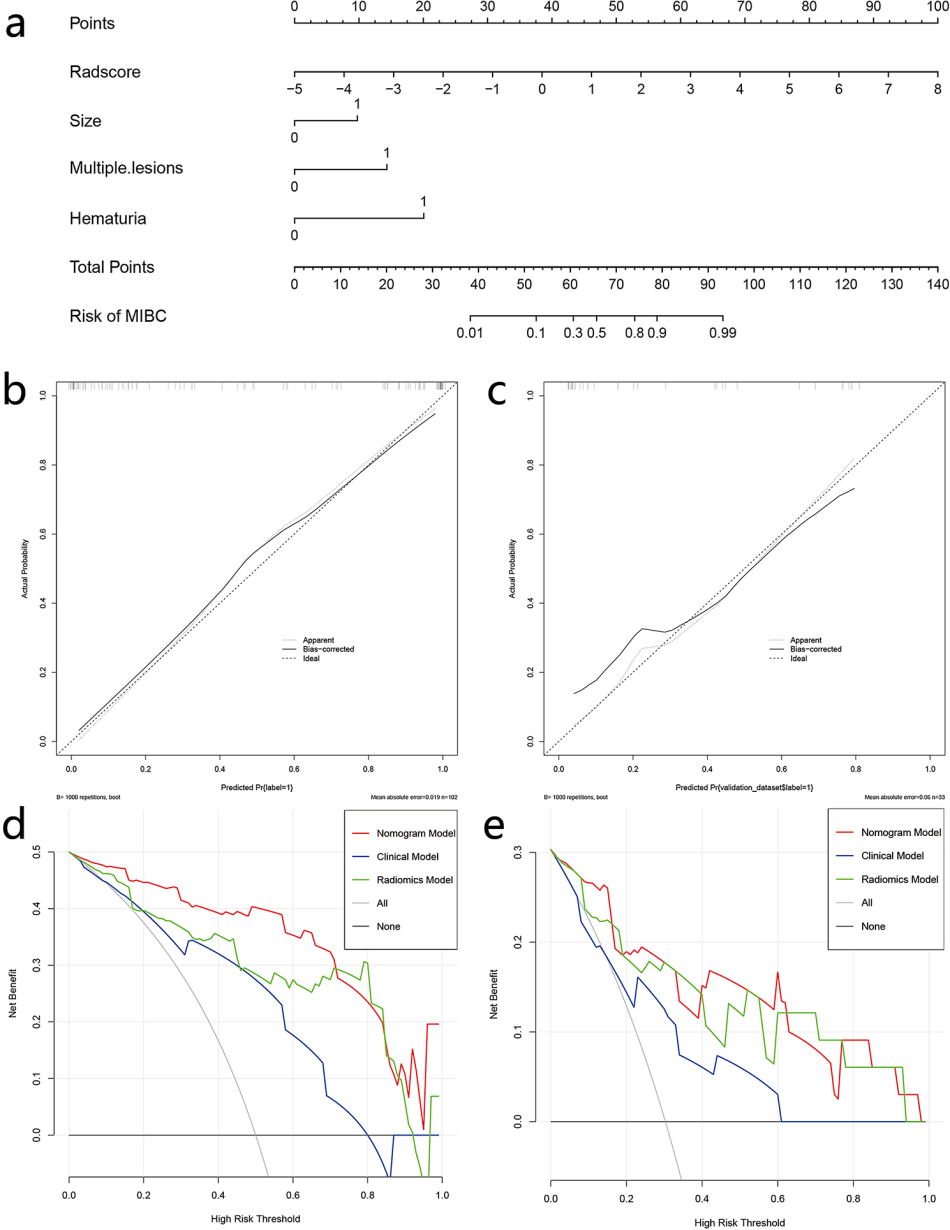
The MRI-based radiomics nomogram for MIBC prediction in patients with BCa **(a)**; Calibration curve of the nomogram in the training **(b)** and validation **(c)** cohorts. DCA for Clinical, Radiomics and Radiomics-Clinical nomogram in the training **(d)** and validation **(e)** cohorts. The y-axis represents the net benefit. The red line represents the radiomics nomogram. The grey line represents the hypothesis that all patients had MIBC

In the validation cohort, the data showed that the Radiomics-Clinical nomogram could better assess preoperative BCa muscle invasion compared to the clinical model alone (AUC = 0.88, AUC = 0.74, *P* = 0.252), but only showed marginal improvement compared to the radiomics model (AUC = 0.88, AUC = 0.87, *P* = 0.853). A comparison of the ROC curves of these three models is shown in Fig. 5. Additionally, the Radiomics-Clinical nomogram exhibited good calibration and favorable clinical net benefit, suggesting it has the potential to become a promising and noninvasive clinical tool for predicting muscle-invasive status (Fig. 6).

### Quantitative analysis of image quality

The results showed that there were no statistically significant differences in SNR and CNR between the 3D-CS-T2-weighted-SPACE and 3D-CS-T2-weighted-SPACE sequences (*P* > 0.05) (Table 4).

**Table 4:** Comparison of SNR and CNR of 3D-T2-weighted-SPACE and 3D-CS-T2-weighted-SPACE sequence images (x ± s)

Finding	3D-T2-weighted-SPACE	3D-CS-T2-weighted-SPACE	*P*
Tumor-SNR	15.6 ± 7.9	14.9 ± 6.3	0.731
Fat-SNR	22.2 ± 12.5	26.0 ± 29.3	0.105
CNR	3.8 ± 3.0	4.1 ± 4.6	0.679

NOTE: SNR, signal-to-noise ratio; CNR, contrast-to-noise ratio

## Discussion

In this study, we developed and validated three sequence models: the 3D-CS-T2-weighted SPACE sequence model, the 3D-T2-weighted SPACE sequence model, and the T2-weighted sequence model, for the preoperative prediction of muscle infiltration in bladder cancer. In recent years, several radiomic models have been developed to assess the muscular infiltration of bladder cancer [12, 20, 29], but most of these models rely on conventional T2WI sequence, which typically feature thicker slices. To our knowledge, this is the first time that CS-SPACE and SPACE have been utilized to construct a radiomics model for evaluating the muscular infiltration of bladder cancer. Our research showed the 3D-CS-T2-weighted-SPACE model exhibited higher AUC value and accuracy compared to the 3D-T2-weighted-SPACE model and conventional T2-weighted model. Moreover, after comparing the SNR and CNR 3D-CS-T2-weighted-SPACE sequence achieved the similar image quality, as 3D-T2-weighted-SPACE sequence while reducing the acquisition time. This indicated that the 3D-CS-T2-weighted-SPACE can provide a more reliable and noninvasive tool for evaluating the depth of bladder tumor invasion preoperatively.

In our study, the 3D-CS-T2-weighted-SPACE model we constructed provided us with rich information extracted from radiomic features, including various features related to wavelet transform and gray-level co-occurrence matrix (GLCM), such as Kurtosis and Skewness under wavelet transform, and the Imc1 feature under GLCM. These features not only offer a deep understanding of image structure and characteristics but also furnish crucial information regarding texture, informativeness, and grayscale distribution during image analysis. Particularly noteworthy is the significant value of these higher-order features in predicting muscular invasion of bladder cancer. By capturing local image features, informativeness, and the skewness of grayscale distribution, these features can more accurately reflect tissue heterogeneity changes caused by muscular invasion of bladder cancer. Compared to first-order features of traditional imaging, these higher-order features provide richer and more detailed image information, thus possessing stronger diagnostic and predictive capabilities, offering robust support for the diagnosis and treatment of muscular invasion of bladder cancer [9, 20].

After the selection based on clinical radiological features, we ultimately included a clinical model composed of three independent risk factors: tumor size, multiple lesions and hematuria. As seen in the selected clinically independent predictors, we found that the maximum tumor diameter size of NMIBC was significantly smaller than that of MIBC (*P* < 0.001), which may suggest that tumors with larger size was more inclined to be MIBC, this clinical factor was similar to that proposed by Zheng et al. [8].

The T2-weighted sequence in bladder examination plays a crucial role in the evaluation of bladder cancer. Standard MRI protocols typically involve acquiring three sets of T2 images in three orthogonal planes perpendicular to the long axis of the bladder. However, obtaining T2-weighted images in three separate planes requires a significant amount of magnet time. The 3D SPACE sequence offers several advantages over its 2D counterpart. Firstly, the 3D SPACE sequence enables acquisition at much higher spatial resolution, especially along the slice direction and without any slice gaps, Qi et al. found that delineating 3D radiomics features of tumors is superior to delineating 2D features, as 3D volume ROIs contain more comprehensive information, thus leading to better diagnostic performance [30]. Secondly, the 3D SPACE sequence produces images that can be easily transferred to the treatment planning system, and the high-resolution images can be reconstructed into any plane without an additional acquisition, making the clinical workflow highly efficient [31, 32]. In our study, because multiplanar reconstruction of coronal and sagittal images is possible, 3D-T2-SPACE can shorten the scan time by approximately 1 min 15 s compared to conventional three-plane T2WI, by incorporating CS technology, the scanning time can be further reduced.

CS is a technique that significantly reduces image acquisition time by under-sampling the k-space. Since its introduction, CS has successfully reduced scan times in various clinical imaging applications [33, 34]. However, before widespread clinical application, there is an important technical challenge to address. Accelerating the image acquisition speed will inevitably affect image quality, and there is no rule to determine to what extent the acquisition speed can be increased without compromising image quality. The optimal acceleration factor required varies for different examination sites, making it difficult to predict the appropriate acceleration factor to be used. Therefore, clinical testing is necessary for validation across various body regions to obtain the optimal acceleration factor.

In our study, to maintain similar image quality between the CS-SPACE and the traditional SPACE sequences, we adjusted multiple parameters of the CS-SPACE, including layer thickness, matrix, and FOV, further reducing the image layer thickness to 0.9 mm. However, the thinner images resulted in an increase in overall data volume and scanning time, offsetting the reduction in data acquisition time achieved by CS technology. Therefore, the effect on reducing the overall scanning time was not significant.

Currently, research on bladder cancer predominantly employs a multi-sequence modality consisting of conventional T2 sequences, along with DWI, ADC, and others [35–37]. Our study’s results demonstrate that 3D T2 sequences with high-resolution thin-slice scanning are superior for diagnosing muscular invasion of bladder cancer compared to conventional T2 sequences. Therefore, incorporating 3D T2WI sequences into the aforementioned multi-sequence modality may provide better diagnostic efficacy for predicting muscular invasion of bladder cancer compared to conventional T2WI sequences alone. Additionally, combining CS can overcome the longer scanning time associated with 3D T2WI scans without compromising image quality. Recent studies have also shown that combining CS with deep learning can further reduce scanning time while achieving better image quality [38]. Khanfari et al. proposed a novel approach called multi-flavored feature extraction or tensor, which suggested that deep features may be more effective than radiomics features alone [39]. The combination of these techniques can offer more significant value for preoperatively predicting muscular invasion of bladder cancer.

However, our study had several limitations. Firstly, the number of cases was insufficient; further research will be conducted with increased sample size. This study was conducted at a single center with internal validation only. Future efforts will involve multicenter studies and external validation. Secondly, we employed the SMOTE technique to equalize the distribution of positive and negative samples, a step that could potentially influence the outcome. Thirdly, we manually outlined the VOI for each tumor region; however, inaccurate manual segmentation might compromise the consistency of feature extraction.

## Conclusions

the 3D-T2-weighted-SPACE sequence incorporation of CS showed better performance for diagnosing muscle invasiveness in BCa and can reducing scanning time. By integrating the Rad-score and clinical indices, the proposed nomogram could enhance the diagnostic performance.

### Electronic supplementary material

Below is the link to the electronic supplementary material.

Supplementary Material 1

Supplementary Material 2

## Data Availability

If there are any further questions or supplementary materials required, I would be more than happy to provide them. You can reach me via email at sure81605@163.com.

## Abbreviations

BCa: Bladder cancer
MRI: Magnetic resonance imaging
3D-CS-T2-weighted-SPACE: 3D-Compressed sensing-T2-weighted-SPACE
CS: Compressed sensing
ICC: Intra-class correlation coefficient
PCC: Pearson correlation coefficient
LASSO: Least absolute shrinkage and selection operator
ROI: Region of interest
VOI: Volume of interest
ROC: Receiver operating characteristic
AUC: Area under curve
NMIBC: Non-muscle-invasive bladder carcinoma
MIBC: Muscle-invasive bladder carcinoma
RC: Radical cystectomy
TURBT: Transurethral resection of bladder tumor
DWI: Diffusion-weighted imaging
ADC: Apparent diffusion coefficient
DCA: Decision curve analysis
NPV: Negative predictive value
PPV: Positive predictive value
CI: Confidence interval
SMOTE: Synthetic minority oversampling technique
SNR: Signal-to-noise ratio
CNR: Contrast-to-noise ratio
