# Supplementary material for: Compressed sensing 3D T2WI radiomics model: improving diagnostic performance in muscle invasion of bladder cancer
Source: BMC Med Imaging. 2024 Jun 17;24:148. doi: 10.1186/s12880-024-01318-0 (PMC11181529; doi:10.1186/s12880-024-01318-0)
Supplement: Supplementary file 1 — Supplementary Material 1 [file 12880_2024_1318_MOESM1_ESM.docx]

**Supporting Figure S1** The process of feature selection using the LASSO algorithm. (a) Selection of the tuning parameter λ in the LASSO classififier via 5-fold cross validation based on minimum criteria. (b) LASSO coefficient profiles of the 5 radiomics features.

**
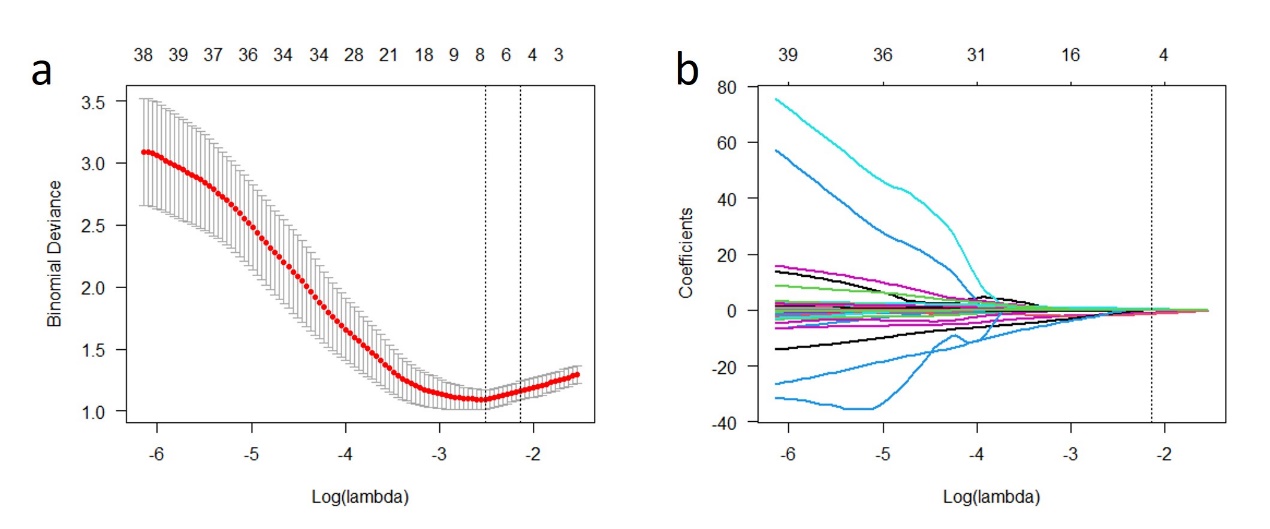
**

**Supporting Table S1** Features and Corresponding Coefficients in the Radiomics Model

|  | Feature |  |  | coef |
| --- | --- | --- | --- | --- |
| 3D-CS-T2-weighted-SPACE | (Intercept) |  |  | -8.9441182 |
|  | wavelet.HLL_firstorder_Kurtosis | |  | 0.3801211 |
|  | wavelet.LLL_glcm_Imc1 | |  | -3.6248021 |
|  | wavelet.HLL_firstorder_Skewness | |  | 1.3055173 |
|  | gradient_firstorder_Kurtosis | |  | 1.7700262 |
|  | log.sigma.1.mm.3D_glcm_Imc1 | |  | -1.6286057 |
| 3D-T2-weighted-SPACE | (Intercept) |  |  | -4.449842 |
|  | log.sigma.1.mm.3D_glcm_Imc1 | |  | -5.510837 |
|  | wavelet.HLH_gldm_DependenceVariance | | | -3.695862 |
|  | wavelet.HLH_firstorder_Kurtosis | |  | 1.795218 |
| T2-weighted | (Intercept) |  |  | -9.253108 |
|  | gradient_glcm_Idn |  |  | 12.09896 |
|  | wavelet.LLH_glszm_SizeZoneNonUniformityNormalized | | | -4.38488 |
|  | squareroot_glszm_LargeAreaHighGrayLevelEmphasis | | | 3.199449 |
