# Supplementary figures and images for: Compressed sensing 3D T2WI radiomics model: improving diagnostic performance in muscle invasion of bladder cancer
Source: BMC Med Imaging. 2024 Jun 17;24:148. doi: 10.1186/s12880-024-01318-0 (PMC11181529; doi:10.1186/s12880-024-01318-0)

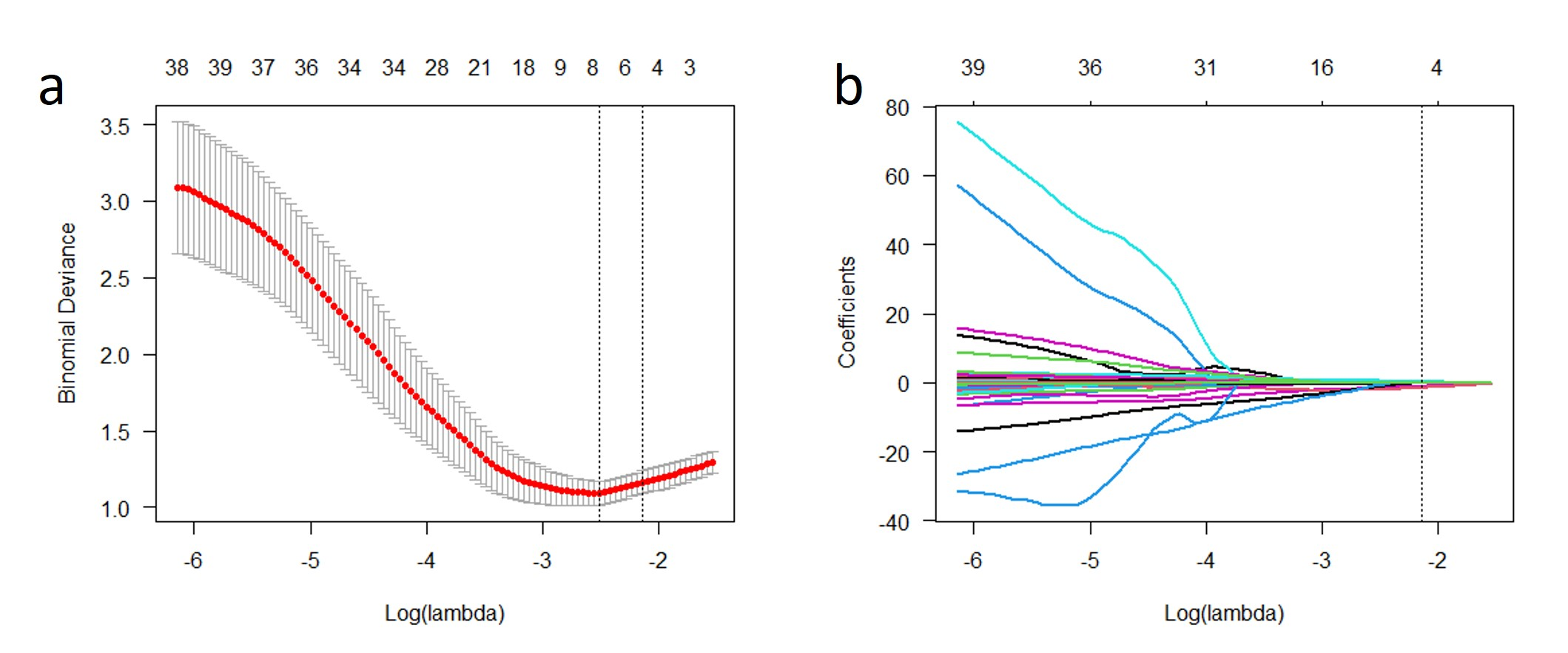

Supplement: Supplementary file 2 — Supplementary Material 2 [file 12880_2024_1318_MOESM2_ESM.png]
